# PreImplantation Trial of Histopathology In renal Allografts (PITHIA): a stepped-wedge cluster randomised controlled trial protocol

**DOI:** 10.1136/bmjopen-2018-026166

**Published:** 2019-01-17

**Authors:** John OO Ayorinde, Dominic M Summers, Laura Pankhurst, Emma Laing, Alison J Deary, Karla Hemming, Edward CF Wilson, Victoria Bardsley, Desley A Neil, Gavin J Pettigrew

**Affiliations:** 1 Department of Surgery, University of Cambridge, Cambridge, UK; 2 NHS Blood and Transplant, Watford, Hertfordshire, UK; 3 Clinical Trials Unit, NHS Blood and Transplant, Cambridge, UK; 4 NHS Blood and Transplant Clinical Studies Unit, Cambridge, UK; 5 Department of Public Health, University of Birmingham, Birmingham, UK; 6 Cambridge Centre for Health Services Research, University of Cambridge, Cambridge, UK; 7 Department of Histopathology, University of Cambridge, Cambridge, UK; 8 Department of Histopathology, University Hospitals Birmingham NHS Foundation Trust, Birmingham, UK

**Keywords:** renal transplantation, transplant surgery, histopathology, stepped-wedge, biopsy, health economics

## Abstract

**Introduction:**

Most potential kidney transplant donors in the UK are aged over 60 years, yet increasing donor age is associated with poorer graft survival and function. Urgent preimplantation kidney biopsy can identify chronic injury, and may aid selection of better ‘quality’ kidneys from this group. However, the impact of biopsy on transplant numbers remains unproven. The PreImplantation Trial of Histopathology In renal Allografts (PITHIA) study will assess whether the introduction of a national, 24 hours, digital histopathology service increases the number, and improves outcomes, of kidneys transplanted in the UK from older deceased donors.

**Methods and analysis:**

PITHIA is an open, multicentre, stepped-wedge cluster randomised study, involving all UK adult kidney transplant centres. At 4-monthly intervals, a group of 4–5 randomly selected clusters (transplant centres) will be given access to remote, urgent, digital histopathology (total intervention period, 24 months). The trial has two primary end points: it is powered for an 11% increase in the proportion of primary kidney offers from deceased donors aged over 60 years that are transplanted, and a 6 mL/min increase in the estimated glomerular filtration rate of recipients at 12 months post-transplant. This would equate to an additional 120 kidney transplants performed in the UK annually. Trial outcome data will be collected centrally via the UK Transplant Registry held by NHS Blood and Transplant (NHSBT) and will be analysed using mixed effects models allowing for clustering within centres and adjusting for secular trends. An accompanying economic evaluation will estimate the cost-effectiveness of the service to the National Health Service.

**Ethics and dissemination:**

The study has been given favourable ethical opinion by the Cambridge South Research Ethics Committee and is approved by the Health Research Authority. We will present our findings at key transplant meetings, publish results within 4 years of the trial commencing and support volunteers at renal patient groups to disseminate the trial outcome.

****Trial registration** number:**

ISRCTN11708741; Pre-results.

**Strengths and limitations of this study**

- Prospective, randomised evaluation of a clinical service using a novel stepped-wedge cluster design which avoids contamination of trial groups, while remaining acceptable to patients and study centres.
- The first national, 24 hours streamlined digital histopathology service, which will support all adult kidney transplant centres in the UK and is likely to have applications beyond the trial setting.
- Example of a low cost ‘registry-based trial’ which uses routinely collected data and has a concomitant economic evaluation to determine cost-effectiveness.
- Use of two primary end points (and therefore nine possible trial outcomes) increases the possibility of an equivocal, rather than definitive result.
- To conserve power, the study will not be stopped early except on the grounds of patient safety.
- Thus, there is a risk that access to histopathology may lead to fewer kidney transplants being performed.

## Introduction

Kidney transplantation is the best treatment for most patients with end-stage renal disease,^1 2^ but shortages in organs mean that patients in the UK wait around 3 years for a transplant.^3^ Over half of the potential pool of deceased donors (people dying in critical care) in the UK are older than 60 years.^4^ Kidneys from donors aged over 60 years have been shown to carry a significantly higher risk of failure than those from younger donors,^5^ and this failure is associated with a substantially increased risk of recipient death.^6^ Consequently, kidneys offered from donors of this age group are less frequently accepted, and once retrieved, are more commonly discarded.^7 8^

### Need for a trial

One approach to assessing which kidneys from older deceased donors (aged >60 years) will provide acceptable long-term function is to perform a biopsy at organ retrieval. The presence of chronic background injury is assessed histologically and its severity can be scored using, for example, the Remuzzi grading system.^9^ Remuzzi has reported that this score correlates with subsequent transplant outcome, and has proposed that, depending on the score the kidney can either be transplanted singly (scores 1–3), discarded (scores≥6) or both kidneys from one donor transplanted into a single recipient (scores 4–5).^9^ One UK transplant centre (Cambridge) has established a preimplantation biopsy service using Remuzzi’s scoring system, but with a modified interpretation of the result. The Cambridge experience demonstrated that kidneys with scores of up to 4 are safe to be used individually, but those with scores of 5–6 should be transplanted together.^10^ Comparison of Cambridge practice to UK transplant activity suggests that this approach resulted in greater numbers of kidney transplants being performed from older donors than would otherwise have been the case.^7^ Outcomes of the transplants were good, in that kidney graft survival from listing was similar to that achieved for all UK deceased kidney transplants—irrespective of donor age.^11^ However, Remuzzi’s approach has not been widely adopted across the UK. One major reservation, which this study will address, is whether biopsy-based selection leads to increased discard rates.^12–14^ In the USA, a large proportion of kidneys are biopsied prior to implantation, and yet discard rates for kidneys from older donors are still high.^15^ In the expectation that preimplantation biopsy analysis is only useful in the subset of deceased donor kidneys in which chronic injury is prevalent, PreImplantation Trial of Histopathology In renal Allografts (PITHIA) will examine transplantation practice for kidneys from deceased UK donors aged over 60 years.

Trial implementation of a national histopathology service is not straightforward. A simple comparison of activity before and after the service is introduced would be unable to distinguish the impact of histopathology from the natural evolution in transplant practice over time. Alternatively, a trial in which individual kidneys are randomised with or without the option to biopsy is not practical. In that study, the availability of extra information for only some kidneys would probably ‘contaminate’ (ie, change) acceptance practice of kidneys offered without it. The pool of elderly deceased kidney donors is already considered high-risk; therefore, implementation of that trial design would be likely to lead to an immediate reduction in acceptance rates for those kidneys offered without a biopsy. The stepped-wedge design addresses these potential problems in two ways. First, the intervention is staggered over the duration of the trial with each centre acting as its own control. Second, at any given centre either all the kidneys will be available to biopsy, or none of them will. This should minimise intracentre contamination effects. Aside from ensuring that the study will have sufficient power to detect differences in the primary outcomes, the stepped-wedge design also has practical advantages over a standard parallel cluster evaluation.^16 17^ The stepped-wedge approach entails that the intervention is rolled out sequentially across the UK, which will allow the trial group time to provide a thorough education programme at each participating centre. This programme will involve a discussion of the indications for, and subsequent interpretation of, the findings of an urgent kidney biopsy. Finally, the design ensures that all centres will eventually gain access to the biopsy service, and patient feedback was strongly in favour of a design which preserved the principle of equity of access.

### Study aims

This paper presents the protocol and rationale for an open, multicentre, stepped-wedge cluster, randomised, registry study. The study will introduce a new, national, histopathology service, and assess the impact of the service on transplant activity and outcomes. A concomitant decision-model based economic evaluation will estimate the lifetime cost-effectiveness of the service.

Specific aims of the study are to:

- Introduce a national histopathology service for transplantation that uses electronic image transfer to rationalise kidney biopsy processing to a limited number of designated biopsy centres.
- Perform a randomised evaluation of this service, by staggering its introduction to all the remaining UK renal transplant centres that do not currently have ready access to 24 hours renal histopathology. The evaluation will adhere to the principles of a stepped-wedge cluster randomised study.
- Determine the cost-effectiveness of this service and thus make recommendations regarding whether it should be continued once the trial is completed, or not.
- Model a low cost randomised registry study that minimises costs because outcome data are collected centrally, which will shape future transplant research.^18^
- This protocol is reported in line with the Standard Protocol Items: Recommendations for Interventional Trials recommendations and has been adapted for publication from the original complete study protocol developed by the Trial Management Group (TMG).^19^

## Methods and analysis

### Description of study design

**Figure 1:**
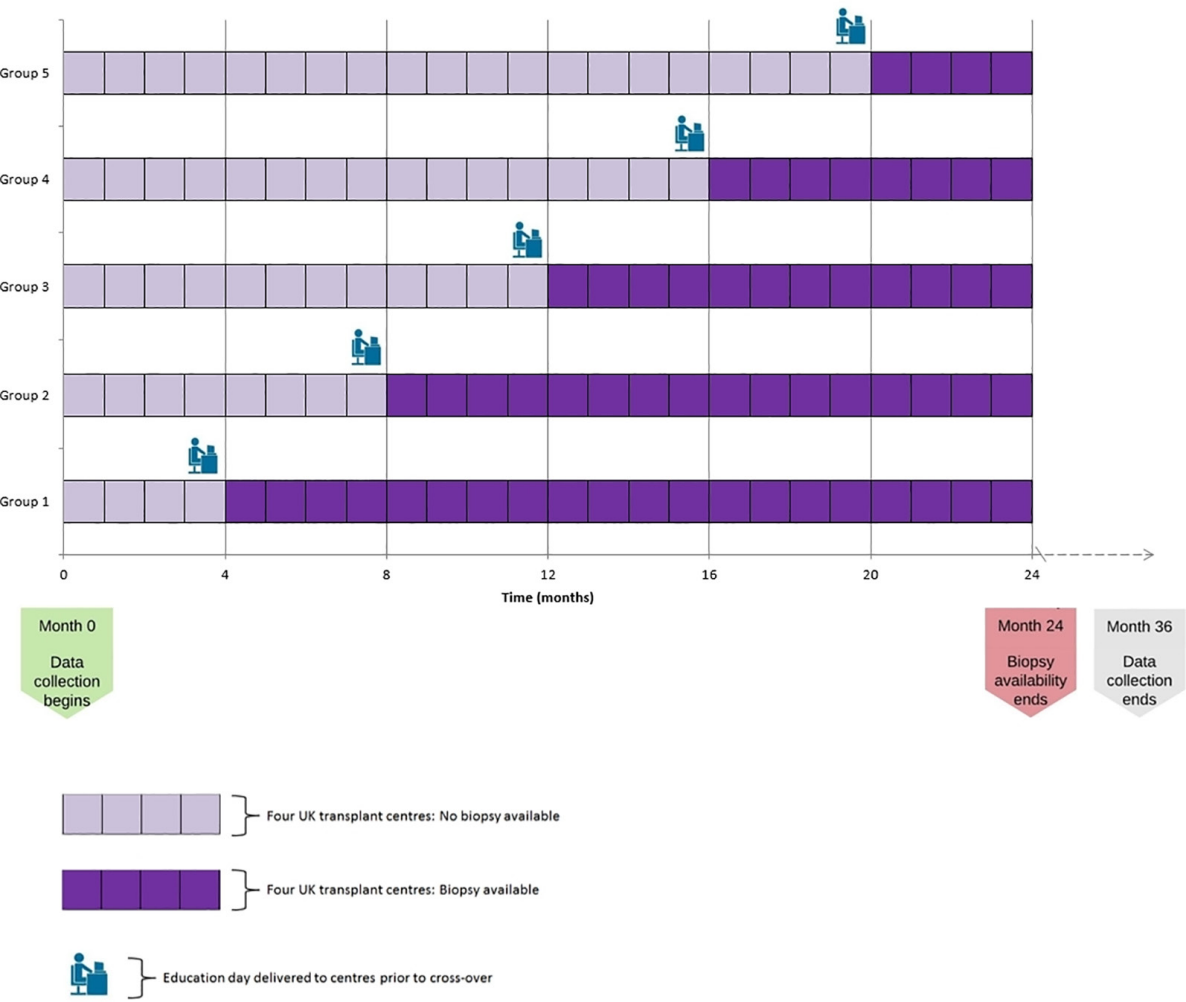
PreImplantation Trial of Histopathology In renal Allografts schema; stepped-wedge cluster randomised design.

There will be 22 UK adult kidney transplant centres (each centre representing one cluster) participating in the randomisation. At 4-monthly intervals, a randomly selected group of four or five clusters will be given access to the new histopathology service. Access will enable clinicians to request preimplantation biopsy analysis as a means of evaluating the suitability of kidneys for transplantation. The overall design is presented in figure 1. The study will compare the number of transplants performed, as well as the outcomes of these transplants before and after each centre gains access to urgent histopathology. Every UK adult kidney transplant centre will participate over the 2 years. Cambridge is involved as one of the biopsy processing centres, although outcomes of transplants performed will not be analysed. Cambridge already supports an urgent preimplantation biopsy service, and is therefore beyond clinical equipoise.

### Study hypotheses

This trial will test the hypothesis: ‘Provision of a national 24/7 preimplantation biopsy service results, at reasonable cost, in transplantation of a greater proportion of kidneys offered from deceased donors aged ≥60 years, and/or improves kidney transplant function at 1-year post-transplant. Numbers of deceased donor kidney transplants performed annually in the UK are consequently significantly increased or improved in quality’.

### Inclusion and exclusion criteria

A biopsy can be requested (at the time of offer) for organs which meet all the inclusion criteria and none of the exclusion criteria. The transplanting surgeon will always decide whether, or not, to biopsy an eligible organ. For the analysis, inclusion criteria are kidneys offered for transplantation from deceased donors (donation after circulatory (DCD) or brain stem (DBD) death) aged ≥60 years. All kidneys offered as a component of a multiorgan transplant are excluded.

### Randomisation

We will use a restricted randomisation technique to randomly allocate the clusters to their cross-over date. To this end, we randomly will select a set of allocation sequences from 10 000 different allocations constrained so that the sum of the total cluster sizes observed under the intervention condition is similar to the total sum of the cluster sizes in the control condition (using historic data). We define ‘similar’ as a difference in the total sums (kidneys) exposed to intervention and control statuses being no different than expected middle 25th percentile range of differences. From this set, one allocation sequence will be selected at random.

Allocation will be performed by an independent statistician once all site approvals are in place and will be revealed to the sites and the trial team approximately 3 months before the date of transition. This approach enables a team of surgeons, renal nephrologists and renal histopathologists to provide a dedicated education package prior to those centres crossing over.

### Intervention and control conditions

#### Control condition

**Figure 2:**
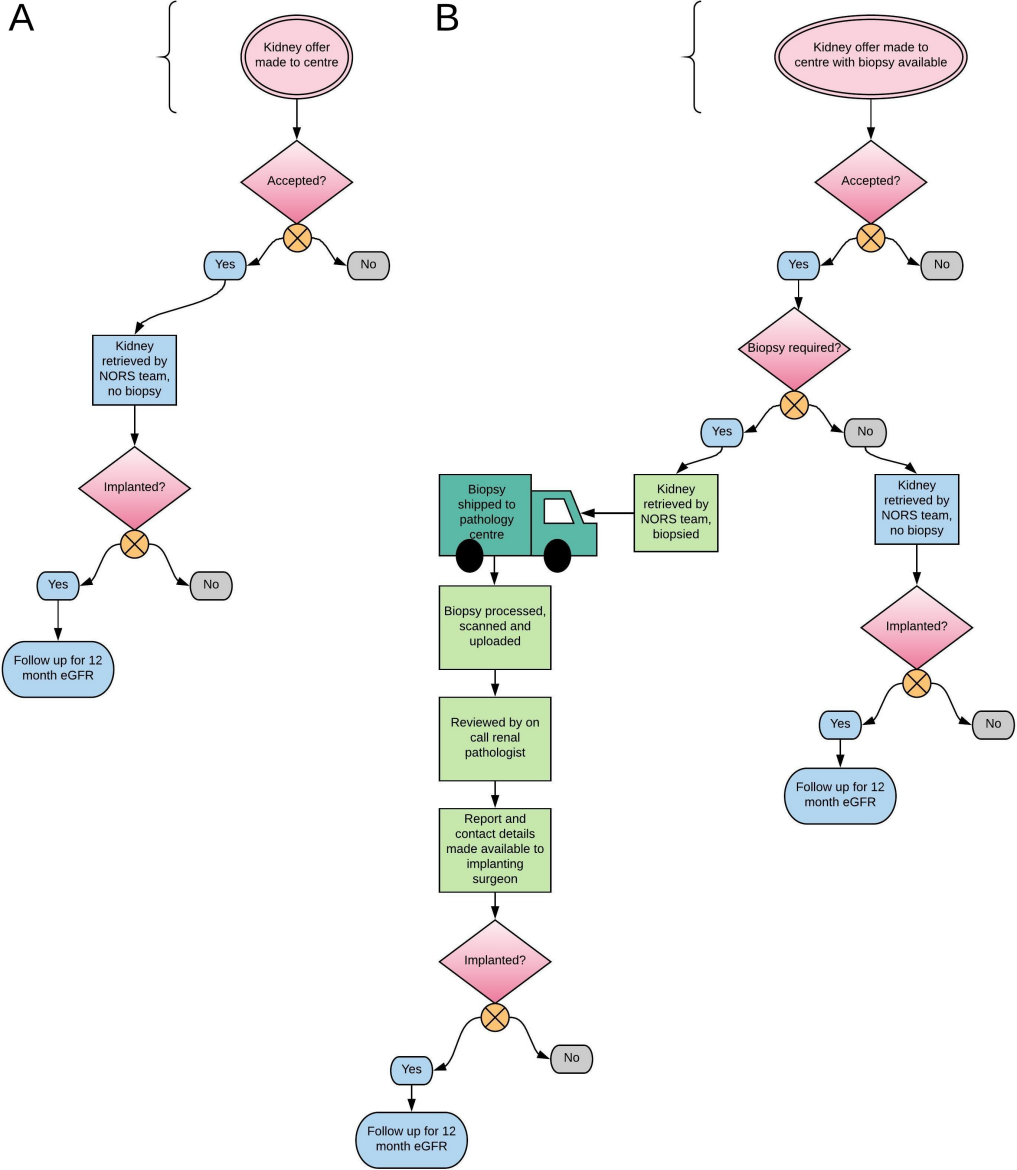
Logistics for biopsies in the control (2A) and intervention (2B) conditions. eGFR, estimated glomerular filtration rate.

UK renal transplant centres, with the exception of Cambridge, do not undertake urgent, preimplantation biopsy analysis. Therefore, the control condition is standard care, which involves accepting and transplanting kidneys from elderly deceased donors, based on donor clinical history and on macroscopic evaluation of the kidney on retrieval (figure 2). On trial commencement, acceptance and transplant activity of all centres will be monitored prior to their accessing the intervention, as this will establish baseline practice.

#### Intervention condition

Once a site has transitioned to the intervention condition they will have access to the national histopathology service (figure 2). Implanting surgeons at those transplant centres can then request a kidney biopsy at the time they are offered a kidney via the UK Transplant Registry held by NHS Blood and Transplant (NHSBT) Hub Operations. Requesting a biopsy is not mandatory—the transplant centres can use the service at their discretion. Transplant centres are also not obliged to adhere to a common set of guidelines governing biopsy use; they, along with their patients, retain the final say over whether a kidney should be biopsied, and/or transplanted.

Patients and their representatives have been involved in the design and implementation phases of PITHIA. Ahead of the trial starting, the TMG have met with key national kidney groups who have offered support for the trial by publicising it in their patient-facing publications. Each participating centre has also received a personalised information sheet to provide patients with an overview of the trial and the intended benefits. Final consent for any transplant will be obtained following a discussion of the potential risks and benefits of the individual kidney on offer, in line with standard practice and national guidelines.

### Biopsy method

A punch biopsy tool (5 mm diameter) will be used to take a sample from the upper pole of the kidney. Areas of the kidney which are scarred will be avoided. The 5 mm punch biopsy will be performed at the retrieval operation by the attending National Organ Retrieval Service (NORS) team and placed in formalin. The biopsy will be transported to the most appropriate histopathology centre, which will be determined on a case-by-case basis, with oversight from NHSBT Hub of Operations. Once the biopsy arrives at the processing centre, the on-call biomedical scientist will process the tissue and embed in wax then cut two sections and stain with H&E as well as Periodic Acid Schiff. They will then scan the slide using a 3DHISTECH (Budapest, Hungary) Panoramic DESK scanner at high resolution (0.12 µm/pixel) and upload the files to a secure, centralised server managed by Sysmex UK (Milton Keynes, England).

At the time of protocol submission, there is one other national study using deceased-donor kidney biopsies (QUOD, http://www.quod.org.uk/index.html). A single 5 mm punch biopsy will provide enough material for both purposes and should be halved longitudinally, maintaining full-depth cortical sampling in both pieces.

### Primary outcome measures

1. Proportion of kidneys aged >60 years that are transplanted on first offer.
2. Estimated glomerular filtration rate (eGFR) measured at 12 months after transplant (acceptable range is between 10 and 15 months after transplant).^20^

### Secondary outcome measures

- Proportion of kidneys used overall (all formal offers for donors aged >60 years, not including fast-track offers).
- Total number of kidney transplants performed.
- Proportion of kidneys discarded after retrieval, out of all retrieved kidneys.
- Number and proportion of ‘single’ and ‘dual’ kidney transplants performed.
- Biopsy utilisation and fidelity, defined as the proportion of kidneys that are biopsied in concordance with the education plan, out of all kidney biopsies.
- Kidney Donor Profile Index of transplants performed.^21^
- Cold ischaemia time, defined as the total time between perfusion of the donor kidneys with cold preservation fluid during retrieval, and reperfusion with recipient blood at implantation.
- 12-month patient survival.
- 12-month graft survival (censored for patient death).
- Proportion of kidneys diagnosed with primary non-function.
- Proportion of kidneys diagnosed with delayed graft function (defined as the use of dialysis during the first postoperative week).

#### Safety outcome measures

- Biopsy-related complication rate.

*Subgroup analyses* (the primary outcome will be replicated for each subgroup):

- DBD donors only;
- DCD donors only;
- Donors who are aged >70 years;
- Centres with historically low median UK Kidney Donor Risk Index of transplanted DCD kidneys (low-risk transplants compared with overall UK activity).

The trial will use data that are routinely collected on the national UK Transplant Registry (UKTR), held by NHSBT. The national registry has a mandatory collection system that produces comprehensive information on organ donors, organs, transplants and recipients.

#### Trial closure

The trial will end 12 months after the final clusters have had access to the histopathology service for 4 months (36 months after the start of the trial). This allows 12-month eGFR to be recorded for all kidneys which are eligible for inclusion in the analysis. The trial has no planned early stopping criteria, although the Trial Steering Committee have the power to stop the trial on safety grounds.

### Sample size calculations

Estimates for the sample size calculations were obtained from the UKTR, held by NHSBT. We followed the methodology proposed by Hooper,^22 23^ and this was implemented using SAS (V.9.4, Cary, North Carolina, USA) and the RShiny WebApp (https://clusterrcts.shinyapps.io/rshinyapp/).

#### First primary outcome: proportion of kidneys that are transplanted on first offer

**Figure 3:**
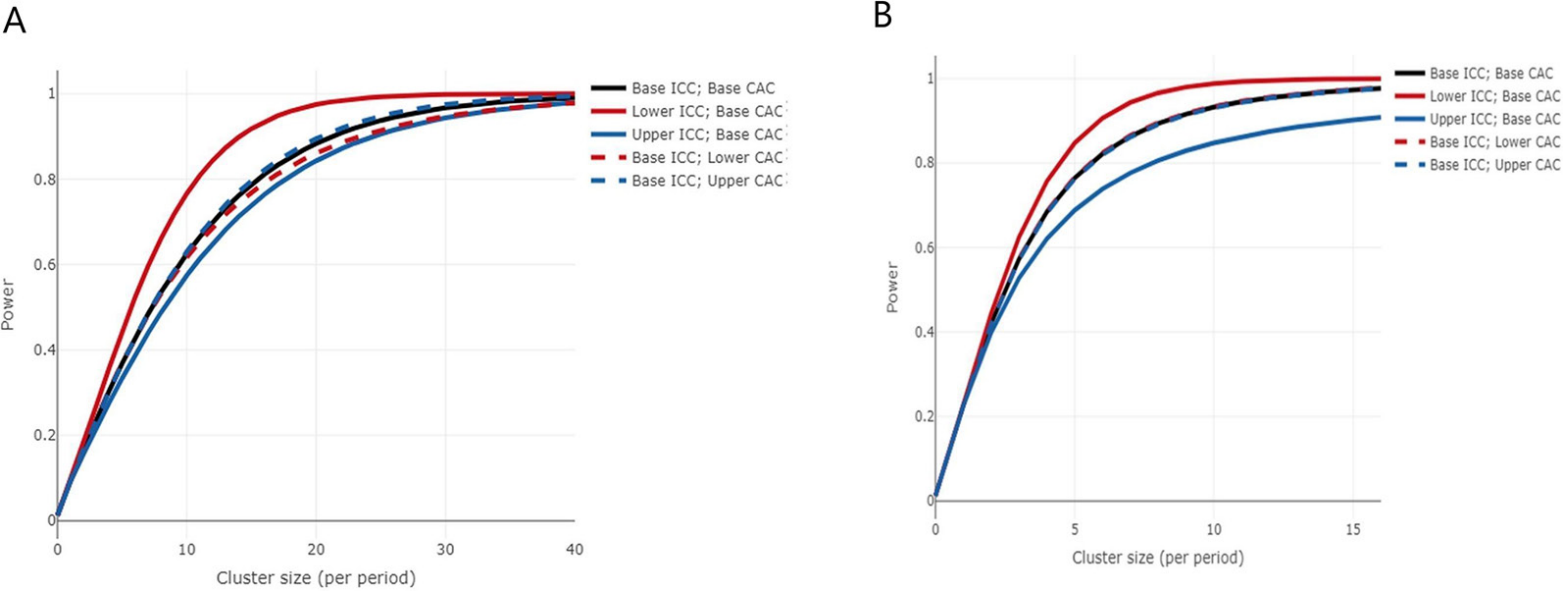
(A) Power curve for the first primary outcome, proportion of kidneys that are transplanted on first offer. The curves show the increase in power as the cluster size increases, for 20 clusters, 5 steps, 4 clusters crossing over at each step and the proportion under control and intervention of 28% and 39%, respectively. The black curve shows the power for the base values of within-period intracluster correlation (WP-ICC) and cluster autocorrelation (CAC) (0.03 and 0.92, respectively), and the remaining curves show for lower and upper levels of the WP-ICC (0 and 0.08, respectively) and CAC (0.74 and 1, respectively). (B) Power curve for the second primary outcome estimated glomerular filtration rate measured at 12 months after transplant. The curves show the increase in power as the cluster size increases, for 20 clusters, 5 steps, 4 clusters crossing over at each step and the mean difference of 6 and SD of 16.06. The black curve shows the power for the base values of WP-ICC and CAC (0.06 and 0.08, respectively), and the remaining curves show for lower and upper levels of the WP-ICC (0.01 and 0.11, respectively) and CAC (0.06 and 0.10, respectively).

Data were extracted on first kidney offers from potential deceased (DBD and DCD) donors aged 60 years and over, in the UK between 1 April 2014 and 31 March 2016. Offers of simultaneous pancreas kidney, multiorgan, fast-track and any offers to Cambridge were excluded. The percentage of first offers which were transplanted was 28.4%. The median number of first offers per cluster per month was 4.38. A mixed linear regression model was fitted to the extracted data, with a binary outcome (whether the offer resulted in a transplant), a fixed effect for date of offer (which was converted into year quarter, ie, six, 4-month time periods called period henceforth), a random effect for cluster and a random interaction between cluster and period. From this model, the within-period intracluster correlation (WP-ICC) and cluster autocorrelation (CAC) were calculated as 0.03 and 0.92, respectively. Assuming an average cluster-period size of 18, to detect a 11% increase in the number of first offers transplanted, the trial will have 85% power (assuming a significance level of 2.5%) and would represent an increase from 28% to 39% acceptance, which we believe would be considered clinically important. We have not allowed for attrition in this calculation as there is unlikely to be any missing data, due to the robustness of the offering data collected by NHSBT. We checked the sensitivity of this calculation by looking at an increase, and separately a decrease, of 2% in the baseline proportion, by varying the WP-ICC (lower WP-ICC set as 0 and upper WP-ICC set as 0.08) and the CAC (lower CAC set as 0.74 and upper CAC set as 1. The trial has within the region of 80% power for all anticipated values of WP-ICC and CAC, and likely values of cluster-period sizes. The power curve, with the baseline proportion set as 28%, is shown in figure 3A.

#### Second primary outcome: eGFR measured at 12 months after transplant

Data were extracted on all recipients who received a kidney only transplant from deceased donors (DBD and DCD) aged ≥60 years, in the UK (excluding transplants at Cambridge) between 1 April 2014 and 31 March 2016. The median number of transplant recipients per cluster per month was 2, hence providing an average cluster-period size of 8. eGFR was calculated using the 4-variable Modification of Diet in Renal Disease (MDRD) formula using uncalibrated serum creatinine measurements,^20^ hence if missing data were present for the recipients sex, age, ethnicity and serum creatinine at 12 months, then eGFR could not be calculated (19%). The mean eGFR was 41.91 with an SD of 16.06.

A mixed linear regression model was fitted to this data, with a continuous outcome (eGFR), a fixed effect for date of transplant (which was converted into year quarter, ie, six, 4-month time periods), a random effect for cluster and a random interaction between cluster and period. From this model, the WP-ICC and CAC were calculated as 0.06 and 0.08, respectively. Assuming an average cluster-period size of 8 (=2×4 clusters), this outcome will have 89% power to detect a mean difference in eGFR of 6 mL/min (assuming a significance level of 2.5%). We checked the sensitivity of this calculation by looking at an increase, and separately a decrease, of 0.5 in the mean difference; and by varying the WP-ICC by ±0.05 and CAC by ±0.016 (which equates to ±20% of the base CAC). The trial has within the region of 80% power for all anticipated values of WP-ICC and CAC, and likely values of cluster-period sizes. The power curve, with the mean difference set as 6, is shown in figure 3B. As the CAC was particularly small, we separately explored the effects of a large CAC value (0.8) and found this had minimal impact.

### Analysis plan

The primary outcome analyses will be performed according to the intention to treat. This will include all eligible patients for whom data have been obtained and will be analysed with respect to the treatment specified by the allocated randomisation order. The primary outcomes will be compared using two-sided tests and at a 2.5% significance level. Departures from randomisation at cluster level are defined as any cluster which does not switch to the intervention when stated in the randomisation order. A cluster is able to withdraw at any point during the trial. Per protocol analysis, which will exclude departures from randomisations and any cluster withdrawals, will also be completed for both primary outcomes of the trial. In situations where a pair of kidneys are transplanted as a ‘dual’ transplant into one recipient (eg, when the biopsy score is >4), this will be counted as half a transplant (in terms of utilisation, not as effective as performing two single transplants, but better than discard).

#### Analysis of primary and secondary outcomes

All primary and secondary outcomes will adjust for calendar time, since the intervention is sequentially rolled out, and cluster, as participants within the same centre are not independent.

The primary aim of the study is to evaluate whether there is a difference in the use of kidneys from older donors before and after exposure to the intervention. For the first primary outcome, a logistic regression model, with random effects for cluster and cluster by period; and adjusting for period (fixed effect), will be used for the hypothesis test and to obtain the associated CI. This will be supplemented with a risk difference obtained from a binomial model with identity link, random effects for centre and centre by period; and adjusting for period (fixed effect). The absolute numbers will be presented by trial arm.

Mean eGFR values in each treatment group at 1 year will be compared using a normal regression model, adjusting for random cluster effect, random cluster by period interaction and a fixed effect for period. As eGFR at 12 months is only recorded for patients who are alive with a functioning graft at 12 months, the primary analysis will be complete case and there will be no imputation for missing values. The number of participants surviving to 12 months will be reported and of those the number who have a 12-month eGFR recorded. The adjusted mean and SD eGFR at 12 months will be reported by treatment arm, alongside the adjusted treatment difference with 95% CI and p value.

A sensitivity analysis will repeat the analysis for the second primary outcome but with the following imputations: if a patient’s graft failed within 12 months, the eGFR at 12 months will be set to the mean value for patients on dialysis in the UK (currently 8.5 mL/min/1.73 m^2^).^24^ If a patient died with a functioning graft within 12 months of transplant, the 12-month eGFR will be set to the mean value at 12 months for all UK transplant patients with a functioning transplant at 1 year (currently 49.4 mL/min/1.73 m^2^). This method assumes that deaths with a functioning graft will occur at random. Multiple imputation was considered futile as insufficient clinical data are collected at the 12-month time point to accurately impute any missing measurements.

#### Other analyses

- Sensitivity analyses: although access to the urgent pathology service is immediate and preceded by formal education, it is still possible that the response (change in behaviour towards older donors) may be more gradual. To monitor this, a sensitivity analysis which excludes the first 2 weeks following crossover to the intervention arm will be conducted, to observe whether this significantly alters our conclusions.
- Variation of effect over time: an assessment of how all centres change their behaviour from the time they are introduced to the urgent biopsy service will be performed. This will be evaluated by estimating treatment effects by time since introduction of the intervention.
- Subgroups: the primary outcome will be replicated for each subgroup separately:
  - DBD donors only;
  - DCD donors only;
  - Donors who are aged >70 years;
  - Centres with a historic low median UK Kidney Donor Risk Index of transplanted DCD kidneys (low risk transplant activity).

Full details of the statistical analyses will be specified in the Statistical Analysis Plan.

#### Health economic analysis

A within-trial and decision model-based economic evaluation will be conducted from the perspective of the National Health Service (NHS), comparing the national histopathology service versus treatment as usual (no biopsy service). The within trial analysis will estimate the cost of the histopathology service including capital investment (eg, scanners), training for centres, staffing and transportation costs, and cost of patient NHS contacts (eg, dialysis, transplant surgery or secondary care contact). Cost data will be extracted from trial records and the UKTR, held by NHSBT. The outcome in the analysis will be the number of patients with a functioning transplant per centre standardised to a 12-month time horizon. The analysis will therefore report the incremental cost per incremental functioning kidney over 12 months.

The within-trial timeframe of 12 months is insufficient to fully capture the differences in costs and outcomes between the study arms. Therefore, a decision model-based economic evaluation will predict demand and supply of kidneys for transplant in the UK, combining cost and outcomes evidence from the trial with other relevant evidence from the literature to determine the incremental cost per quality-adjusted life-year gained from the service. Full details of the economic analyses will be specified in the Health Economics Analysis Plan.

### Patient and public involvement

Kidneys from donors aged over 60 years have been shown to carry a significantly higher risk of failure than those from younger donors. Transplant patients therefore face a dilemma at listing, ‘should I only accept kidneys from young healthy donors, but wait longer in doing so, or should I also accept kidneys from older, more marginal donors?’ Since 2014, Cambridge has discussed at listing, whether recipients are willing to accept kidneys from higher risk donors, including older donors. The central question of PITHIA—whether transplant rates from older donors can be increased with the use of preimplantation biopsy analysis—requires an underlying willingness from patients to accept these organs. In Cambridge, 75% (124 of 169) of our listed population consented to kidneys from older donors, suggesting that this would also be accepted nationally among the wider transplant patient population.

Success of the trial is critically dependent on ongoing patient involvement, because informed consent to receive an older kidney cannot be obtained without an appreciation of the associated risks and how these are potentially offset by biopsy analysis. During the design of the study, the trial group had lengthy discussions regarding key research questions, trial design and dissemination of results with patients. The proposed study design was presented at the National Kidney Advisory Group and Renal Transplant Services Meetings (which have patient and public involvement representation), with additional involvement of two of Cambridge renal transplant patients (one of whom is a co-applicant and sits on the steering group). These discussions shaped the development of the trial in several ways. In addressing the basic research question, patients felt that the particularly high rates of discard of kidneys from elderly DCD donors were difficult to justify. They also raised concerns relating to the inequity of access to transplantation between different UK renal transplant centres and felt strongly that the wide variation in waiting times for deceased donor transplantation were unjustifiable.^3^ Their opinion was that this variation is at least partly due to differences in DCD kidney transplant practice and welcomed attempts (as in the current study) to improve transplant yields in this group. Patient concerns regarding inequity of access to kidney transplantation also had a major influence in trial design. We had originally proposed a conventional cluster randomised trial, in which half of the transplant centres were given access to the urgent biopsy service, and the other half continued as before, without access. However, the patients felt strongly that it was unfair to deprive some patients access to a service that may potentially increase their chance of transplantation. As a result, we opted instead for a stepped-wedge cluster approach, because this way, the histopathology service is made available eventually to all centres.

Kidney patient groups have been extremely supportive of the trial and PITHIA has been publicised in multiple patient facing publications (including Kidney Life and Hope Kidney Patient’s Association). Additionally, the trial group continue to engage with the public through the study’s website (www.pithia.org.uk), its twitter account (@PITHIA_trial) and by publishing non-scientific summaries in local media outlets (eg, Cambridge evening news, centre for evidence in transplantation, Cambridge NIHR biomedical research centre and Blood and Transplant matters).

## Discussion

This study will examine whether the introduction of a National Digital Pathology Service for urgent preimplantation kidney biopsy analysis increases the number of kidney transplants performed in the UK; or improves their outcomes. In doing so, the study will address the persisting controversy regarding the use of biopsies to decide which kidneys should be implanted singly, as part of a dual transplant or discarded.^12–14 25 26^ A successful study outcome—that kidney transplant numbers are increased, and the quality of grafts is maintained—would provide strong support that biopsy features correlate with long-term transplant function, and that the biopsy can be used as a predictive tool to inform transplantation decisions. However, there are several reasons why, despite a correlation with preimplantation biopsy analysis and transplant outcome, the trial may fail to show an increase in kidney transplant numbers. These include National Offering of a ‘marginal’ kidney from an elderly donor for a named recipient who is unsuitable for such a kidney—typically a very young recipient. In addition, the biopsy service will be introduced for use at the clinicians’ discretion, and either inappropriate biopsy use, or disregard of biopsy findings may prejudice trial outcomes. Thus, to some extent, the trial tests the willingness of the UK kidney transplant body to adopt and observe biopsy practice.

Failure to provide biopsy results to clinicians promptly, reliably and safely all pose risks to service uptake after roll-out. The punch biopsy method was chosen because of its reliability, and to avoid the limitations of the more commonly used ‘needle core’ and ‘surgical wedge’ techniques. The needle core is small, and it often contains insufficient numbers of glomeruli (25) and arteries (1) for Remuzzi assessment.^27 28^Second, the maximum depth of the needle is difficult to appreciate, particularly in a flaccid, non-perfused kidney. As a result, the core frequently samples medulla (not useful for analysis) and risks the deeper structures within the kidney. Alternatively, in performing the surgical wedge biopsy, the surgeon uses a scalpel to resect an ellipse of renal cortex. The quality of the sample is extremely variable, because there are no anatomical landmarks or standard measuring tools to guide the size or the depth of the sample. Additionally, when performing the ‘wedge’, there is generally a bias towards a superficial, subcapsular resection, which avoids damaging deeper structures but fails to sample the deep cortical tissue that is considered most representative of the ‘quality’ of the kidney graft. Wedge biopsies, partly because of their shape, tend to oversample the superficial cortex and overestimate the amount of glomerulosclerosis because of the predominance of sclerosis in the subcapsular region.^27^ In contrast, the punch biopsy tool sets the dimensions of the sample, so it provides a consistent, representative sample of renal cortex (equal volumes of superficial and deep cortex, more reliable sampling of arteries) without risking the deeper structures which are protected by the bevel which limits insertion to safe depth (8 mm).^29^ Once the sample is taken, it will immediately commence formalin fixation at the donor hospital, so that on arrival at the processing centre it should be ready for rapid processing (4 hours), thereby minimising any potential impact on the cold ischaemia time.

For the PITHIA study, a consortium of 15–20 voluntary consultant renal histopathologists from around the country will remotely assess and score the biopsies for severity of chronic injury (by grading glomerulosclerosis, tubular atrophy, interstitial fibrosis and arterial narrowing), and relay their assessment to the relevant transplant clinicians. The use of digital whole slide images avoids the need for the pathologist to be on-site to assess the glass slides, which requires an on-call rota at each centre—difficult to man with specialists. Thus, an important consequence of a national rota is the ability to efficiently provide a specialist on-call rota, with only one renal pathologist on-call for the whole country at any given time, with the option of a second backup pathologist to assist if busy or to provide a second opinion in difficult cases. Additionally, the trial group will provide training, feedback and discussion, allowing the pathologists to converge on common thresholds of grading, minimising interobserver variability. This will continue to be monitored during the trial.

PITHIA is an example of an effective, registry-based randomised trial, in which follow-up data are routinely collected as part of the mandatory post-transplant UKTR held by NHSBT. This has multiple advantages for the patients and the centres who are monitored following the intervention. Patients will avoid extra appointments, tests and the added bureaucracy generally associated with clinical trial participation. Similarly, once the trial has begun, transplant centres (other than those six centres providing the biopsy service) will have almost no extra work related to the trial. Compared with non-registry trials, data collection costs will be minimised, while participation is maximised.^18^ This saves substantially on trial costs.

## Ethics and dissemination

This trial complies with the Declaration of Helsinki (2013). It will also be conducted in compliance with the approved protocol, the principles of Good Clinical Practice, the UK Data Protection Act, the requirements of the Human Tissues Act and the National Health Service Research Governance Framework for Health and Social Care. The trial has been prospectively registered with the ISRCTN (ISRCTN11708741).

We expect to present our findings to allied transplant professionals as oral presentations at key transplant meetings in the UK and abroad. We are also committed to publishing the results within 4 years of the trial commencing, in a journal compliant with Open Access. Any publication will coincide with local and national press release. If effective and cost-effective, we will consult with relevant NHS bodies to continue provision of the service. Volunteers from each of the renal patient groups attached to the various UK transplant centres will be supported in disseminating the trial results to the waiting list population at each centre, and if proven effective and cost-effective, we hope to involve local kidney patient groups in the implementation of changes in local practice which follow on from this study.

## Supplementary Material

Reviewer comments
