# Supplementary material for: PreImplantation Trial of Histopathology In renal Allografts (PITHIA): a stepped-wedge cluster randomised controlled trial protocol
Source: BMJ Open. 2019 Jan 17;9(1):e026166. doi: 10.1136/bmjopen-2018-026166 (PMC6340453; doi:10.1136/bmjopen-2018-026166)
Supplement: Reviewer comments [file bmjopen-2018-026166.reviewer_comments.pdf]

PEER REVIEW HISTORY

BMJ Open publishes all reviews undertaken for accepted manuscripts. Reviewers are asked to complete a checklist review form (**http://bmjopen.bmj.com/site/about/resources/checklist.pdf**) and are provided with free text boxes to elaborate on their assessment. These free text comments are reproduced below.

ARTICLE DETAILS

**TITLE (PROVISIONAL)**	Pre-Implantation trial of Histopathology In renal Allografts (PITHIA): A stepped-wedge cluster randomised control trial protocol
**AUTHORS**	Ayorinde, John; Summers, Dominic; Pankhurst, Laura; Laing, Emma; Deary, Alison; Hemming, Karla; Wilson, Edward; Bardsley, Victoria; Neil, Desley; Pettigrew, Gavin

This article was not externally reviewed at BMJ Open. Protocols that have been independently assessed prior to submission to BMJ Open are usually fast-tracked to publication on the grounds that further substantial changes will not be possible. This independent assessment will usually be external, independent review for both a substantial grant award from a non-commercial or government funder and ethics approval.
